# Experiences of nutritional management in patients with breast cancer-related lymphedema: a qualitative study guided by Fitch's supportive care framework

**DOI:** 10.3389/fnut.2026.1961839

**Published:** 2026-10-23

**Authors:** Ming Yu, Chunfang Li, Yangyang Cao, Yizhou Gu, Xiaolan Zhou, Xu Zhang, Ronghui Geng, Dandan Gu

**Affiliations:** 1 Department of Nursing, Affiliated Rudong Hospital of Xinglin College, Nantong University, Nantong, China; 2 Department of Nursing, Nantong Tongzhou Hospital of Traditional Chinese Medicine, Nantong, China; 3 Department of General Surgery, Nantong Tongzhou Hospital of Traditional Chinese Medicine, Nantong, China

**Keywords:** breast cancer, fitch theory, lymphedema, nursing care, nutritional management

## Abstract

**Purpose:**

To explore patients' experiences, perceptions, self-management practices, and unmet supportive care needs regarding nutritional management in breast cancer-related lymphedema, and to inform future patient-centered interventions.

**Methods:**

Guided by Fitch's Supportive Care Framework, we developed a semi-structured interview guide and conducted in-depth interviews with 13 patients with breast cancer-related lymphedema recruited via purposive sampling between January and October 2025. Interview data were analyzed using a framework-guided hybrid inductive–deductive thematic analysis. This approach integrated theory-driven coding informed by Fitch's framework with data-driven theme development.

**Results:**

Three core themes emerged, encompassing nine sub-themes: (a) inadequate understanding and misconceptions regarding nutrition management, comprising lack of lymphedema-specific nutritional knowledge, poor recognition of diet-disease associations, and low self-efficacy in nutritional management; (b) practical challenges in nutrition management, comprising lack of nutritional management skills, fluctuations in dietary adherence, ambiguity in attributing symptoms to dietary causes, and absence of a self-monitoring system; and (c) gaps in support systems and unmet needs, comprising insufficient family collaboration and support and lack of continuous professional guidance.

**Conclusions:**

Patients with breast cancer-related lymphedema described multifaceted difficulties in nutritional management across cognitive, behavioral, and support dimensions. These findings suggest that healthcare providers should recognize these real-world experiences when planning supportive care. Future interventions may consider combining cognitive support, skill-building, and systematic support; however, this qualitative study cannot determine whether such interventions affect lymphedema outcomes or self-management capabilities.

## 1 Introduction

Breast cancer is the most prevalent malignant tumor affecting women worldwide, with continuously rising incidence and improved long-term survival owing to continuous advancements in multimodal treatment (1, 2). Despite favorable survival outcomes, breast cancer-related lymphedema (BCRL) remains a common, irreversible, and debilitating chronic complication after breast cancer surgery and radiotherapy, affecting 20%−30% of breast cancer survivors (3, 4). As a lifelong chronic condition, BCRL requires sustained and standardized self-management to control limb swelling, reduce infection risks, and maintain upper limb function and daily living ability (5–8).

Complete decongestive therapy and compression garment intervention are well-recognized core interventions for BCRL clinical management (9, 10). In recent years, nutritional management has been increasingly discussed as a potentially modifiable supportive care component within BCRL self-management (11). Some quantitative and mechanistic studies have suggested that weight control, anti-inflammatory dietary patterns, and nutritional status regulation may be associated with systemic inflammation and lymphedema-related symptoms. However, causal associations with, or therapeutic effects on, BCRL progression remain uncertain and warrant further investigation (12, 13). Nevertheless, current clinical guidelines for BCRL nutritional management lack targeted, standardized, and stage-specific recommendations (14). Most existing guidance offers generalized dietary advice with inconsistent and even contradictory recommendations, failing to accommodate the individualized needs of BCRL patients (15, 16).

Furthermore, existing studies have predominantly adopted quantitative designs to examine associations between dietary factors and lymphedema severity (17, 18). In contrast, there is limited evidence capturing patients' lived experiences, personal perceptions, self-management practices, and unmet supportive care needs from a patient-centered perspective. Many patients with BCRL adjust their daily diet based on personal experience in the absence of standardized professional guidance. Nevertheless, the perceived benefits, adherence challenges, and long-term self-management patterns associated with these self-initiated dietary adjustments remain understudied (19).

Fitch's Supportive Care Framework provides a systematic theoretical model for analyzing patients' dynamic supportive care needs throughout the entire cancer illness trajectory, covering informational, practical, emotional, and social needs in different disease stages (20). This framework has been widely applied to explore supportive care needs in various cancer populations (21, 22), yet it has not been used to systematically interpret the phase-specific challenges and multidimensional needs of nutritional management in BCRL patients.

Against this background, this qualitative study adopted Fitch's Supportive Care Framework as the theoretical guidance to explore the lived experiences and unmet supportive care needs of nutritional management among women with BCRL. The findings aim to fill the research gap in patient-centered BCRL nutritional supportive care, provide theoretical and practical evidence for developing targeted, staged nutritional intervention strategies, and ultimately improve patients' nutritional self-efficacy, symptom control, and overall quality of life.

Against this background, this qualitative study was guided by Fitch's Supportive Care Framework to explore the lived experiences, personal perceptions, self-management practices, and unmet supportive care needs related to nutritional management among women with BCRL. The findings aim to fill existing gaps in patient-centered nutritional supportive care for BCRL and inform the development of targeted, stage-specific nutritional support strategies. Importantly, this study does not evaluate the clinical efficacy of nutritional management or its causal effects on lymphedema progression. Instead, it captures patient-reported insights from real-world experiences to support future intervention design and hypothesis generation.

### 2.1 Study design

This qualitative descriptive study explored the experiences, perceptions, self-management practices, and unmet supportive care needs associated with nutritional management among patients with breast cancer-related lymphedema. Fitch's Supportive Care Framework guided the full research process and offered a structured lens to interpret patients' dynamic needs and adaptive behaviors over the disease trajectory. We applied a framework-guided hybrid inductive–deductive thematic analysis to systematically code, categorize and develop core themes from interview data. The design, conduct and reporting of this study adhered to the COREQ checklist (23).

### 2.2 Participants and setting

We recruited participants from the Breast Oncology Department and Lymphedema Rehabilitation Clinic of a tertiary hospital in eastern China between January and October 2025. Using purposive maximum variation sampling, we sought maximum variation across age, BMI, education level, employment status, monthly income, marital status, time since cancer diagnosis, and duration of BCRL.

Inclusion criteria included the following: (1) pathologically confirmed breast cancer and clinically diagnosed BCRL for at least 3 months; (2) reported actively adjusting their diet in an attempt to manage lymphedema-related symptoms; (3) age of 18 years or older and ability to communicate fluently in Chinese. Exclusion criteria were as follows: (1) comorbid conditions requiring strict dietary restrictions, such as severe hepatic or renal insufficiency or uncontrolled diabetes mellitus; (2) cognitive, psychiatric, or auditory impairments that could interfere with study participation; (3) recurrent or metastatic (Stage IV) breast cancer. All eligible participants provided written informed consent prior to enrollment.

### 2.3 Recruitment and enrollment

Recruitment was conducted jointly by clinical staff and research team members. Attending physicians and specialist nurses first screened medical records of inpatients and outpatients to identify potential candidates. Trained researchers then approached eligible patients in person to explain the study purpose, content, interview format, and significance.

Patients who expressed initial interest underwent a one-on-one secondary screening to confirm their willingness and eligibility. Those who agreed to participate received detailed written information and signed consent forms. We then scheduled interviews at flexible times and private locations according to participants' preferences. A recruitment log was maintained to document screening outcomes, enrollment status, and reasons for non-participation.

### 2.4 Ethical considerations

This study was approved by the institutional review board of a tertiary hospital in eastern China [Ethics Approval No. (2025)20]. All procedures followed the ethical principles of the Declaration of Helsinki. Before data collection, participants received verbal and written explanations of the research purpose, procedures, potential risks and benefits, and privacy protections. All participants provided voluntary written informed consent.

Interviews were conducted in private, quiet rooms to protect confidentiality. Participants were informed that participation was voluntary, that they could refuse any question or withdraw at any time, and that this would not affect their clinical care. All identifiable information was anonymized during data processing and analysis.

### 2.5 Data collection

We conducted semi-structured, face-to-face individual interviews. Three trained researchers (two postgraduate nursing students and one psychological counselor) conducted the interviews. All had prior experience in qualitative interviewing and cancer patient communication. Before data collection, they received standardized training on interview techniques, framework application, and handling of unexpected situations.

The semi-structured interview guide was developed based on Fitch's Supportive Care Framework (Table 1). Interview questions corresponded to the core domains of the framework. These domains included Feeling Known, which explored participants' understanding of nutrition and information needs, Managing Daily Living, which covered daily dietary practices and practical challenges, and Navigating the System, which addressed professional and social support. The guide also created space for participants to share emotional and psychosocial experiences linked to nutritional management. This same framework guided the deductive stage of data analysis, maintaining consistency between data collection and analysis. Prior to each interview, researchers restated the principles of anonymity and confidentiality and obtained participants‘ consent for audio recording.

**Table 1:** Interview guide based on Fitch's supportive care framework.

Fitch core domain	Corresponding supportive care needs	Interview questions
Feeling known	Care information needs; Psychological/affective needs	1. What channels do you use to obtain nutrition management advice? Is this information clear and practical enough? 2. What dietary guidance support would you most like to receive that is not currently available? 3. Does dietary control cause psychological stress for you? How do you cope with it?
Managing daily living	Physiological needs; Rehabilitation and daily care needs; Psychological/affective needs	1. Have you actively adjusted your dietary nutrient intake to manage lymphedema? What specific measures have you implemented? What challenges have you encountered? 2. Are there any foods that make you feel better or worse? 3. How do you balance nutritional needs and edema control during the rehabilitation process? 4. What are the main challenges of maintaining nutrition management in daily life? 5. Do your dietary habits change during emotional fluctuations? How does this affect symptoms?
Navigating the system	Psychosocial needs	1. Do your family or friends support your diet management? How does their attitude affect you? 2. How do you cope with dietary restrictions in social situations?

Interviews lasted 28 to 65 min. Researchers used flexible probing based on participants' responses, observed non-verbal cues (facial expressions, tone, gestures), and took field notes. Data collection continued until thematic saturation, defined as two consecutive interviews yielding no new themes or insights, as independently confirmed by two researchers.

At the end of each interview, participants completed a questionnaire covering basic personal information and clinical details, including age, BMI, education level, employment status, monthly income, marital status, time of cancer diagnosis, and duration of BCRL.

### 2.6 Data analysis

We used a framework-guided hybrid inductive–deductive thematic analysis, combining theory-driven classification with data-driven theme development. Two researchers independently performed the analysis and cross-checked each other's work, following these steps: Step 1: Data preparation and immersion. All interviews were transcribed verbatim in Chinese within 24 h. Researchers read the transcripts while listening to the audio recordings to correct errors and gain familiarity with the data. Step 2: Deductive initial coding. Using NVivo 12, researchers independently coded the transcripts. Guided by Fitch's three core dimensions, which are Feeling Known, Managing Daily Living and Navigating the System, they deductively categorized relevant statements into preliminary codes. This framework was also used to structure the interview guide, so the deductive coding categories corresponded to the domains explored during interviews, while inductive analysis allowed additional themes to emerge from the data. Step 3: Inductive theme development. Within each deductive category, researchers inductively sorted, compared, and summarized the data to extract sub-themes and core themes that reflected participants' actual experiences, including findings not anticipated by the framework. Step 4: Theme integration and consensus. The two researchers compared their coding results and thematic structures. Discrepancies were resolved through iterative discussions, with senior team members consulted when consensus could not be reached, to produce a final unified thematic framework.

### 2.7 Trustworthiness

We applied Lincoln and Guba's criteria, namely credibility, dependability, confirmability, and transferability, to ensure rigor throughout the study. Credibility was enhanced through member checking (approximately 30% of participants reviewed and confirmed the preliminary themes), regular multidisciplinary peer debriefing, and theoretical triangulation using Fitch's framework. Dependability was achieved by maintaining a detailed audit trail of all key decisions (sampling, interviews, coding, theme revisions) and through stepwise replication, in which two researchers independently coded the data and reconciled their results. Confirmability was addressed through researcher reflexivity, meaning all researchers documented their prior assumptions and potential biases before and during analysis, and by providing thick descriptions with original verbatim quotes to create a transparent link between raw data and final themes. Transferability was supported by comprehensive reporting of the study context, sampling strategy, participant characteristics, and methodological procedures, enabling readers to assess the applicability of the findings to similar populations and settings.

## 3 Results

The study initially recruited 18 eligible women with BCRL. Five participants withdrew during the study owing to scheduling conflicts or changes in health status, leaving a final sample of 13 female participants who completed in-depth interviews. Thematic saturation was reached with this sample. Participants had a mean age of 44 years (range: 26–67 years). Time since breast cancer diagnosis ranged from 9 to 30 months, and BCRL duration varied across individuals. All participants had finished primary cancer treatment, were in the stable phase of lymphedema management, and reported actively adjusting their diet in an attempt to manage lymphedema-related symptoms. This qualitative study did not assess the impacts of these dietary adjustments on lymphedema outcomes. Detailed demographic and clinical characteristics of participants are presented in Table 2.

**Table 2:** Demographic and clinical characteristics of participants (*n* = 13).

Participant ID	Age (years)	BMI category	Education level	Employment status	Monthly income (CNY)	Marital Status	Time since breast cancer diagnosis (months)	Duration of BCRL (months)
A	52	Normal weight	Lower secondary school	Employed	3,000	Married	10	8
B	50	Normal weight	Lower secondary school	Employed	3,000	Married	16	10
C	67	Normal weight	No formal education	Unemployed	2,000	Divorced	14	10
D	42	Normal weight	Lower secondary school	Employed	3,500	Married	30	22
E	45	Normal weight	Primary school	Unemployed	4,000	Married	25	15
F	30	Normal weight	Junior college	Employed	7,500	Married	17	12
G	47	Normal weight	Lower secondary school	Employed	3,500	Married	16	7
H	59	Normal weight	Primary school	Unemployed	4,000	Married	10	3
I	42	Normal weight	Upper secondary school	Employed	4,500	Married	9	9
J	43	Overweight	Lower secondary school	Unemployed	3,000	Divorced	25	9
K	26	Overweight	Upper secondary school	Employed	6,600	Married	30	7
L	35	Obese	Junior college	Unemployed	3,500	Married	9	3
M	29	Normal weight	Bachelor's degree	Employed	8,000	Married	11	5

BMI, body mass index; BCRL, breast cancer-related lymphedema; CNY, Chinese yuan. BMI categories were defined according to Chinese criteria: normal weight < 24.0 kg/m^2^; overweight 24.0–27.9 kg/m^2^; obese ≥ 28.0 kg/m^2^. Monthly income is presented in Chinese yuan (CNY).

Analysis of interview data using a framework-guided hybrid inductive–deductive thematic analysis generated three core themes and nine corresponding sub-themes describing the experiences of patients with BCRL regarding nutritional management. Drawing on Fitch's Supportive Care Framework, these themes map onto different dimensions of supportive-care needs: (a) inadequate understanding and misconceptions regarding nutrition management (aligning with Feeling Known: information-seeking and need for validation), with sub-themes of lack of lymphedema-specific nutritional knowledge, poor recognition of diet-disease associations, and low self-efficacy; (b) practical challenges in nutrition management (aligning with Managing Daily Living: integrating diet into everyday life), with sub-themes of skill deficits, fluctuating adherence, symptom-cause ambiguity, and absence of self-monitoring; and (c) gaps in support systems and unmet needs (aligning with Navigating the System: accessing professional and social resources), with sub-themes of insufficient family collaboration and lack of continuous professional guidance. An overview of the thematic structure is presented in Table 3.

**Table 3:** Themes and sub-themes.

Themes	Sub-themes
Inadequate understanding and misconceptions regarding nutrition management	Lack of lymphedema-specific nutritional knowledge
	Poor recognition of diet-disease associations
	Low self-efficacy in nutritional management
Practical challenges in nutrition management	Lack of nutritional management skills
	Fluctuations in dietary adherence
	Ambiguity in attributing symptoms to dietary causes
	Absence of a self-monitoring system
Gaps in support systems and unmet needs	Insufficient family collaboration and support
	Lack of continuous professional guidance

### 3.1 Theme 1: inadequate understanding and misconceptions regarding nutrition management

Although nutritional management is often discussed as an important supportive strategy for BCRL, participants frequently described it as a source of confusion and distress. This confusion and distress arose from limited foundational knowledge, persistent misconceptions about diet–disease relationships, and conflicting information from various sources.

#### 3.1.1 . Sub-theme 1: lack of lymphedema-specific nutritional knowledge

Participants generally understood the value of a “healthy diet,” yet lacked concrete, actionable knowledge tailored to lymphedema self-management. Knowledge gaps included recognition of hidden dietary sodium, the role of high-quality protein, and how different foods influence fluid balance and systemic inflammation.

“Sometimes I think I'm eating lightly, but then I'm told there's salt in noodles and bread too—I'm totally baffled. It's impossible to guard against everything!” (Participant D)“Everyone says to eat more protein. But will eating meat and soup make my edema worse? How much is actually okay?” (Participant F)“I heard watermelon and mung beans help with water retention. Can I eat them? Will they cause any problems?” (Participant C)

#### 3.1.2 Sub-theme 2: poor recognition of diet-disease associations

Many participants perceived dietary effects on lymphedema as delayed and non-specific, making it difficult to attribute swelling episodes to particular food choices. Consequently, some underestimated dietary risks and believed occasional dietary lapses would not worsen lymphedema.

“Edema tends to come and go anyway. It's hard to say for sure that today's flare-up is caused by yesterday's pickles.” (Participant M)“You can't avoid everything forever. I think as long as you're careful most of the time, an occasional slip-up shouldn't hurt.” (Participant H)“The first time I broke my rule, I felt guilty. But when nothing happened, I got bolder and my guard came down.” (Participant A)

#### 3.1.3 Sub-theme 3: low self-efficacy in nutritional management

Participants commonly reported low self-efficacy for sustaining long-term, complex dietary modifications they believed were needed for lymphedema management. They expressed doubts about their ability to follow dietary recommendations and were prone to abandoning dietary goals when confronted with cravings or practical difficulties.

“For others, eating is pure enjoyment. For me, it's like solving math problems—constantly calculating and strategizing. It's exhausting. I'm just not cut out for this.” (Participant L)“Sticking with it for a week or two is manageable, but forever? I honestly don't have the confidence.” (Participant B)“Sometimes I give in to a craving, then regret it. I feel like such a failure—I have zero willpower.” (Participant K)

### 3.2 Theme 2: practical challenges in nutrition management

Even when participants received nutritional advice for lymphedema, translating recommendations into real-world daily life imposed substantial burdens. These practical difficulties stemmed from everyday living constraints, personal preferences, and social contexts.

#### 3.2.1 Sub-theme 1: lack of nutritional management skills

Participants reported deficits in practical skills required for nutritional self-management, including low-sodium cooking practices, interpreting food nutrition labels, and planning balanced daily meals consistent with lymphedema-related advice.

“I added just a tiny bit of salt, but you can't taste any saltiness at all. It feels like I can't even swallow the rice.” (Participant H)“When I go to the supermarket, what on the packaging counts as high sodium and what counts as low? I just don't get it.” (Participant J)“The biggest headache every day is figuring out what to eat. I keep going over the same few options, worried about making the wrong choice or not getting enough nutrition.” (Participant I)

#### 3.2.2 Sub-theme 2: fluctuations in dietary adherence

Consistent dietary adherence was difficult to maintain over time. Taste preferences, emotional states, and social-family dining contexts frequently led to lapses and compromises in dietary practice.

“After a lifetime of rich, flavorful food, now you want me to eat bland, watery soup every day? It's so tasteless that I can barely manage it. When I can't stand it anymore, I just give in.” (Participant G)“When I'm in a bad mood, I just can't care less. Eating what makes me happy comes first.” (Participant K)“Everyone else at home eats regular meals. They can't cook special meals just for me every day. After a while, I just started eating with them.” (Participant E)

#### 3.2.3 Sub-theme 3: ambiguity in attributing symptoms to dietary causes

Participants struggled to disentangle whether limb-swelling fluctuations stemmed from diet, physical activity, or other factors. Such uncertainty hindered their capacity to adjust self-management strategies appropriately.

“Sometimes I'm careful about what I eat and I'm not even tired, but my arms still swell up. It's so baffling.” (Participant F)“Since we can't pinpoint the cause, we don't know what to do next. Should I be stricter with my diet, or get more rest? We're just fumbling in the dark.” (Participant L)

#### 3.2.4 Sub-theme 4: absence of a self-monitoring system

Few participants had structured routines to track dietary intake alongside changes in limb swelling. Without such personalized feedback, nutritional management largely relied on trial-and-error.

“I know it's good to keep track, but how exactly do I do it? Should I measure my arms daily, or just eyeball it? And how should I record what I eat?” (Participant E)“When I feel really bloated, I look back at the past few days and realize I've tried a little bit of everything. But what actually caused it? I have absolutely no clue.” (Participant I)

### 3.3 Theme 3: gaps in support systems and unmet needs

Although nutrition is increasingly recognized as an important component of supportive care for BCRL, participants experienced a notable mismatch between this acknowledged importance and the practical, ongoing support accessible to them. Their need for individualized, sustained nutritional guidance was inadequately met by current healthcare and social resources.

#### 3.3.1 Sub-theme 1: insufficient family collaboration and support

Family members often lacked adequate understanding of BCRL-specific dietary requirements, leading to limited practical collaboration around meal-preparation. At times, family-based traditional eating expectations created extra psychological burden for participants.

“They always think I'm dieting or being picky, not realizing this directly affects whether my arms swell up.” (Participant M)“I can't eat spicy food, but my family loves it. I can't expect them to cater to me every day, but cooking for myself makes me feel isolated.” (Participant D)“They think my meals are too bland and lacking in nutrition, always urging me to eat more ‘good stuff.' But what they consider ‘good' is precisely what I dare not eat in excess.” (Participant J)

#### 3.3.2 Sub-theme 2: lack of continuous professional guidance

Following hospital discharge, participants had limited access to long-term, personalized nutritional counseling. Without easy-to-access channels to consult dietitians or specialist nurses, many felt left to manage this complex condition independently and expressed strong demand for convenient ongoing professional support.

“Now I run into new issues like constipation or loss of appetite, and I don't know who to ask. Should I adjust my diet? How should I adjust it?” (Participant G)“Follow-up appointments are so short. The doctor mainly checks for tumor recurrence and has no time to ask about my diet or edema.” (Participant A)“I can't keep going back to the hospital for every little thing. If only I had a nutrition consultant like a family doctor I could consult online.” (Participant B)

## 4 Discussion

Guided by Fitch's Supportive Care Framework, this qualitative study identified three interrelated challenges affecting nutritional self-management among women with BCRL: cognitive limitations, practical implementation difficulties, and insufficient systemic support. These challenges correspond to three key domains of the framework: Feeling Known, Managing Daily Living, and Navigating the System. They may help explain why patients with BCRL struggle to translate professional dietary guidance into long-term, consistent behavioral change. All findings are based on patient-reported experiences. This study cannot establish whether nutritional management prevents, alleviates, or modifies BCRL progression. The following sections discuss each challenge in relation to existing evidence and outline implications and hypothesis-generating directions for future intervention development.

### 4.1 Cognitive barriers: knowledge that doesn't translate (feeling known)

Participants generally recognized the value of healthy dietary habits yet lacked concrete, actionable guidance and felt uncertain about their ability to maintain long-term adherence. This common knowledge-behavior discrepancy is well established in chronic illness (24–26). In the BCRL context, this gap presented distinct characteristics. Participants did not clearly understand how dietary factors such as sodium intake, protein consumption, and fluid balance might influence limb swelling, and they found it difficult to establish a clear connection between daily diet and subsequent physical changes in the affected extremity. Consistent with this finding, Zhu et al. (27) reported that unclear associations between dietary behavior and disease status may undermine patient self-management motivation. Such persistent uncertainty appeared to gradually weaken participants' self-confidence. Participants frequently expressed self-doubt regarding their self-management capacity, describing themselves as unable to persist in dietary adjustments and as lacking sufficient self-discipline. These subjective perceptions suggested not only limited professional health knowledge but also a reduced sense of personal agency in rehabilitation.

Why does current education fail to address this? Much of current education emphasizes restriction: avoid salt, avoid fat, avoid this and that. Participants described these messages as generating anxiety rather than capability. They expressed a need for practical, contextual knowledge, including how to season food without salt, how to read a label, and how to eat out. They also described needing small wins: achieving a modest goal, seeing a peer succeed, or noticing a change. Future education may therefore need to pivot from “what to avoid” to “how to manage,” and use incremental goals and peer modeling to help rebuild the belief that dietary change is possible and worthwhile.

### 4.2 Practical implementation: daily life gets in the way (managing daily living)

Even with clear knowledge of recommended dietary adjustments, participants still encountered substantial barriers to consistent adherence. Salt-free cooking did not align with habitual dietary practices, and interpreting nutrition labels remained an unfamiliar skill. Additionally, tailoring personal meals to meet perceived BCRL-related dietary requirements while accommodating family eating preferences imposed a considerable logistical burden. Participants also reported fluctuating motivation throughout their self-management journey. They were able to follow dietary guidelines on some days, yet struggled to maintain adherence on other days because of food cravings or negative emotional states. Fluctuating limb swelling further complicated their self-management efforts, as participants found it difficult to distinguish potential triggers. They were unable to attribute swelling changes to specific factors, such as a high-sodium diet, housework, or variable weather conditions. Such ambiguity in symptom attribution is not unique to BCRL and has been documented across multiple chronic illnesses with multifactorial and fluctuating symptoms (28).

Participants' narratives indicate that health education alone may be insufficient to support sustainable dietary self-management, suggesting the potential value of practical, skill-based components in future programs. Group cooking sessions have been reported to enhance dietary competence and self-confidence among individuals with chronic diseases, yet the applicability of such strategies to the BCRL population remains underexplored (29). Beyond hands-on training, participants also expressed a strong need for simplified monitoring tools to systematically record daily dietary intake, lifestyle behaviors, and changes in limb symptoms. Paper-based or mobile-enabled daily logs may help transform vague subjective feelings into structured, actionable health information. Symptom tracking has been reported to be effective for trigger identification and self-management in other chronic conditions, although its value for BCRL rehabilitation requires further empirical verification (30, 31). As participants emphasized, they recognized the benefits of regular monitoring but lacked clear operational guidance for implementation. Structured health diaries may reduce speculative judgment in daily self-care and strengthen patients' perceptions of behavioral control, even though their direct effects on lymphedema-related outcomes remain unconfirmed.

### 4.3 Systemic support: no one to call (navigating the system)

Lymphedema is a lifelong chronic condition, yet professional nutritional support declines substantially following hospital discharge. Participants in this study described two interconnected gaps in long-term rehabilitation support. First, they perceived that many family caregivers lacked adequate understanding of the dietary adjustments they believed were related to BCRL. Second, they described clinical teams as having no standardized follow-up protocols or dedicated time to sustain ongoing nutritional guidance after treatment completion. These perceived gaps were often described as creating interpersonal tension during daily meal preparation. Some family members dismissed dietary adjustments as unnecessary, while others encouraged unrestricted intake of richly flavored foods that participants perceived as potential triggers for limb swelling. When new health concerns emerged, including constipation, reduced appetite, or unexplained worsening of lymphedema, participants reported lacking accessible professional channels for timely guidance and problem-solving support.

Although these supportive care gaps are not exclusive to BCRL, they may be particularly consequential given the lifelong nature of lymphedema self-management. Addressing these gaps may benefit from attention at both familial and systemic levels. Within household settings, family caregivers may benefit from clear, practical information about the dietary adjustments patients perceive as relevant to BCRL management and about ways to provide support without inducing guilt or psychological pressure. Clinicians could also acknowledge the social challenges of sustained dietary modification and help patients view occasional dietary lapses as common, rather than focusing on strict adherence alone. At the institutional level, hospitals could consider strengthening rehabilitative continuity through multidisciplinary teams that include dietitians and lymphedema specialists (32, 33). Supplementing in-person consultations with remote follow-up via telehealth platforms or mobile applications may help address post-discharge support gaps and reduce patients' sense of being left without professional guidance between visits.

### 4.4 Strengths and limitations

This study has three strengths. The application of Fitch's framework provided a structured approach to organize patient narratives rather than offering simple descriptive summaries. In-depth interviews captured nuanced experiential details that would not be accessible through survey methods. Furthermore, maximum-variation sampling recruited a diverse sample of participants and improved the transferability of the study findings.

This study has several limitations that warrant consideration. First, participants were recruited from a single hospital in eastern China. This sampling strategy may restrict the transferability of findings to BCRL patients in other regions, clinical settings, or healthcare systems. Second, the cross-sectional design captures participants' experiences and supportive care needs only at one time point. Fitch's Supportive Care Framework highlights that supportive care needs evolve across the illness trajectory; accordingly, this study cannot describe longitudinal changes in patients' nutritional self-management challenges and needs. Third, this study was not designed to examine whether dietary modification alters the course, severity, or progression of BCRL. As a qualitative cross-sectional investigation, it cannot establish causal relationships or evaluate any therapeutic effects of nutritional management on BCRL. Fourth, the analysis primarily relies on patient self-reported accounts and does not include complementary perspectives from clinicians or family caregivers. Integrating these stakeholder viewpoints would support the development of practical, patient-centered interventions. In addition, self-reported dietary behaviors are vulnerable to recall bias and social desirability bias. However, iterative probing during interviews and detailed field notes partially mitigated these methodological limitations. Future research may adopt longitudinal designs to track shifts in self-management needs, incorporate multi-stakeholder perspectives, and explore the feasibility and potential value of the supportive care directions generated from this study.

### 4.5 Implications for practice and research

For clinical practice, participants' narratives point to three potential priority areas. First, future care may need to shift from generic dietary guidance to BCRL-relevant, skill-focused education. Second, patients may benefit from simple self-monitoring tools, such as paper diaries or mobile applications, to help them observe patterns among diet, daily behaviors, and limb symptoms. Third, healthcare systems could consider establishing sustained support through telehealth check-ins, family education sessions, and multidisciplinary clinics. These practice implications aim to inform future intervention design and hypothesis generation, and they do not confirm the clinical efficacy of such approaches.

For research, four potential directions merit consideration. Longitudinal studies could track how patients' nutritional needs and barriers change over time. Mixed-methods designs could quantify how common each barrier is and which matters most. Future intervention studies could examine the multi-component support strategies suggested by these findings. Finally, future work could include socioeconomic and cultural factors to identify which groups may be most underserved.

## 5 Conclusion

This qualitative study reveals that BCRL patients encounter intertwined cognitive, practical, and systemic barriers throughout nutritional self-management. Participants reported inadequate actionable knowledge, persistent difficulties in daily behavioral implementation, and insufficient supportive resources from both family members and clinical providers. Aligned with Fitch's Supportive Care Framework, optimal supportive care needs to cover three core domains. Corresponding to the domains of Feeling Known, Managing Daily Living, and Navigating the System, future interventions should deliver targeted and operational health information, cultivate practical daily self-care skills, and establish sustainable family and healthcare support networks, respectively. These findings offer directional implications for future intervention design, without providing confirmatory evidence that nutritional management alters BCRL onset or progression.

Our single-center, cross-sectional design limits transferability and precludes causal or therapeutic inferences regarding nutritional management in BCRL. Nevertheless, these findings offer a patient-centered foundation for developing and evaluating comprehensive nutritional support programs. This study does not claim that dietary modification alters lymphedema outcomes; rather, it aims to deepen understanding of patients' experiences and unmet support needs and to inform more responsive care for women living with BCRL.

## Data Availability

The qualitative interview datasets generated during this study are not publicly available due to containing information that could compromise participant privacy. Anonymized transcripts may be available from the corresponding author upon reasonable request and subject to a formal data sharing agreement.
